# Reduction in promotor methylation utilizing EGCG (epigallocatechin-3-gallate) restores RXRα expression in human colon cancer cells

**DOI:** 10.18632/oncotarget.9204

**Published:** 2016-05-06

**Authors:** Jay Morris, Vondina R. Moseley, April B. Cabang, Katie Coleman, Wei Wei, Elizabeth Garrett-Mayer, Michael J. Wargovich

**Affiliations:** ^1^ Department of Molecular Medicine, University of Texas Health Science Center at San Antonio, San Antonio, TX 78229, USA; ^2^ Department of Cell & Molecular Pharmacology, Medical University of South Carolina, Charleston, SC 29425, USA; ^3^ Department of Public Health Science, Medical University of South Carolina, Charleston, SC 29425, USA; ^4^ Department of Biostatistics & Epidemiology, Medical University of South Carolina, Charleston, SC 29425, USA

**Keywords:** RXRα, EGCG, methylation, epigenetics, colon cancer

## Abstract

Silencing of regulatory genes through hypermethylation of CpG islands is an important mechanism in tumorigenesis. In colon cancer, RXRα, an important dimerization partner with other nuclear transcription factors, is silenced through this mechanism. We previously found that colon tumors in Apc^Min/+^ mice had diminished levels of RXRα protein and expression levels of this gene were restored by treatment with a green tea intervention, due to reduced promoter methylation of RXRα. We hypothesized that CIMP+ cell lines, which epigenetically silence key regulatory genes would also evidence silencing of RXRα and EGCG treatment would restore its expression. We indeed found EGCG to restore RXRα activity levels in the human cell lines, in a dose dependent manner and reduced RXRα promoter methylation. EGCG induced methylation changes in several other colon cancer related genes but did not cause a decrease in global methylation. Numerous epidemiological reports have shown the benefits of green tea consumption in reducing colon cancer risk but to date no studies have shown that the risk reduction may be related to the epigenetic restoration by tea polyphenols. Our results show that EGCG modulates the reversal of gene silencing involved in colon carcinogenesis providing a possible avenue for colon cancer prevention and treatment.

## INTRODUCTION

Genetic mutations have long been a central theme in the causality of cancer. Recently, Hanahan and Weinberg [1] expanded their previous tenets on the origins of human cancer to now include epigenetic events in the pathway of carcinogenesis. Across the paradigm of cancer, recent data suggest that epigenetic events are of central importance in regulation of tumor formation and progression, possibly creating a new avenue for prevention and treatment [1–3]. There are many intrinsic and extrinsic factors that encompass epigenetic changes and these can involve diet, heritability and the environment. Regulation of the epigenome is under the control of DNA methyltransferases (DNMTs), histone deacetylases (HDACs), histone acetyltransferases (HATs) and associated modifier proteins [2, 4, 5]. Compounds regulating these proteins or altering their function is an emerging field for drug development. Some pharmacologic inhibitors such as 5-Aza-2′-deoxycytidine (5aza-dc) and suberoylanilide hydroxamic acid (SAHA) have entered clinical testing, but off-target toxicity has limited its progress [6].

Targeting epigenetic regulators is a new paradigm for cancer prevention [6–9]. Within this field small molecule natural products (SMNPs) such as resveratrol (from grapes) and genistein (from soy) are reported to modulate cancer risk while modifying epigenetic pathways [7, 10]. One extensively studied SMNP is epigallocatechin gallate (EGCG), the major polyphenolic compound from green tea [8, 11–17]. This compound has many pleiotropic effects in cancer prevention, attributable to its antioxidant, anti-inflammatory, and cell signaling properties. In the epigenetic channel, EGCG has been shown to inhibit DNMT function leading to a reduction in promoter methylation [18, 19]. While there are reports of EGCG inhibiting experimental models of breast, liver, colon, pancreatic, and prostate cancers, the influence of EGCG on epigenetic pathways has been a relatively underexplored route of chemoprevention [12, 14–17, 20–24]. Earlier, we reported that the nuclear transcription factor RXRα was epigenetically silenced in the AOM/Apc^Min/+^ mouse model for colon cancer [13], yet its expression was restored with a green tea intervention. The consequence of a dysfunctional RXRα was recently high-lighted in two clinical studies where polymorphisms in this gene were associated with proximal colon cancer [25, 26]. Impairment of RXRα functionality may be a fundamental mechanism for tumor progression. RXRα is a nuclear transcription factor from the retinoid family. It is involved in numerous signaling pathways due to its ability to heterodimerize with other transcription factors such as liver X receptor (LXR) [27], farnesyl X receptor (FXR), retinoic acid receptor (RAR), peroxisome proliferator-activated receptor (PPAR) and vitamin D receptor [28]. Taken together, current research suggests that RXRα is a potential critical regulatory element in colon cancer.

Loss of RXRα either by genetic mutation or epigenetic regulation in mice underscores its importance in colon cancer development and makes it a vital nexus of control for incipient tumors to circumvent [29]. To further clarify this relationship we utilized human tissue microarrays to establish whether RXRα expression is downregulated and human colon cell lines to determine if EGCG epigenetically modifies the methylation status of the RXRα promoter. We found loss of RXRα protein in human colon cancers and cell lines. Treatment of colon cancer cells with EGCG restored RXRα protein levels and reduced the amount of promoter methylation, revealing more evidence for EGCG as an epigenetic regulator in colon cancer chemoprevention.

## RESULTS

**Figure 1:**
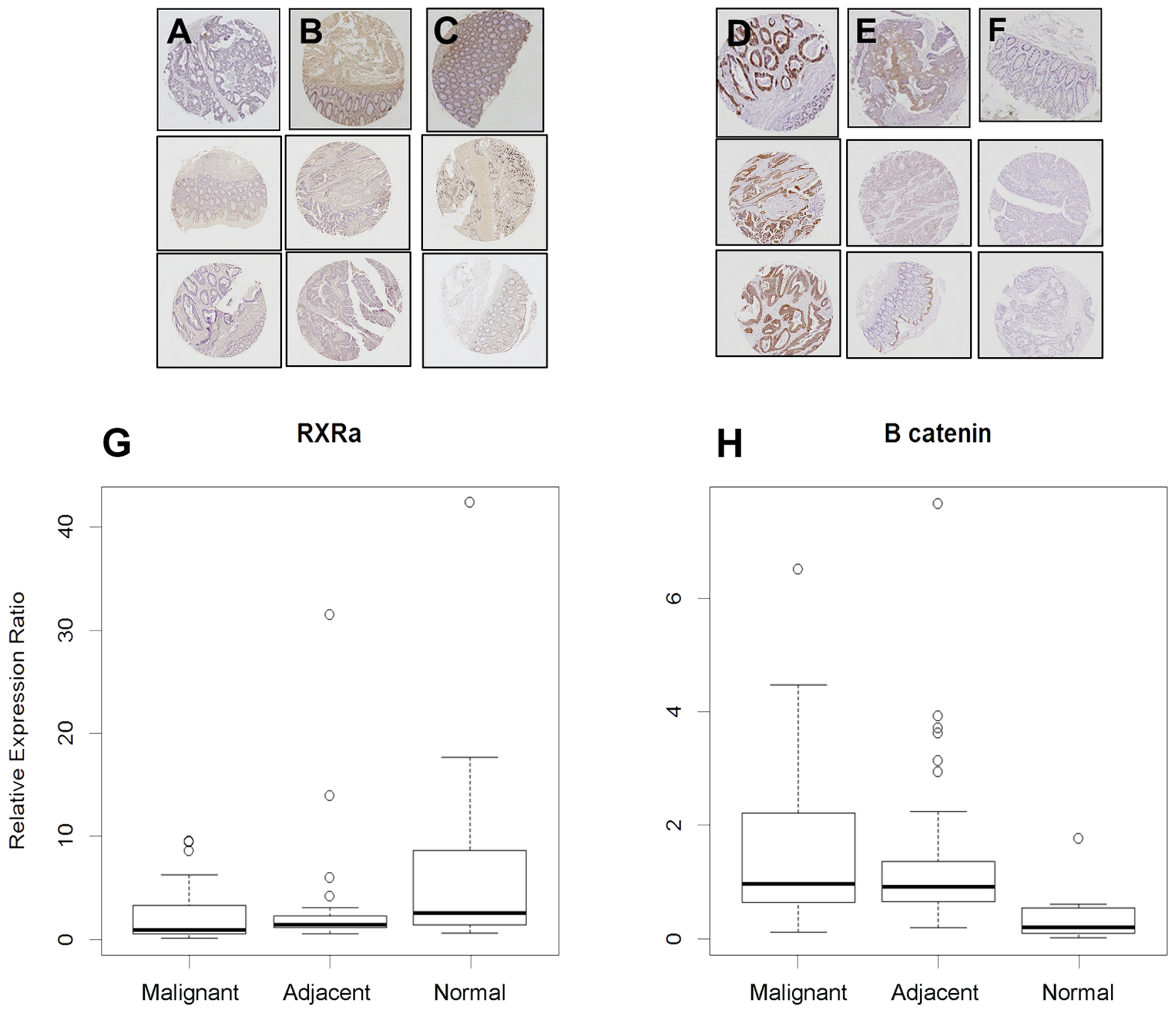
RXRα and β-catenin immunostaining in human tissues **A.** RXRα staining is notably absent in human colon tumors, but present in **B.** adjacent and **C.** normal colon mucosa (*p-values: normal vs. adjacent = 0.029, normal vs malignant = 0.0035, adjacent vs malignant = 0.23) while **D.** β-catenin staining is high in human colon tumors and in **E.** adjacent tissues but low in **F.** normal colon mucosa. **G.** Quantitated expression of RXRα and **H.** β-catenin in various tissues, data displayed as box and whisker plots (black line – mean, circles outliers, box 50% mean distribution and lines 99% distribution) (*p-values: normal vs adjacent = 0.001, normal vs malignant = 0.0005, adjacent vs malignant = 0.48).

Our earlier report that RXRα expression is silenced in the AOM/Apc ^Min/+^ mouse model for colorectal cancer [13] prompted an exploratory analysis of RXRα protein expression in human colorectal cancers and established colorectal cancer cell lines. To assess RXRa expression in human colorectal cancers we used a commercial tissue microarray and stained for RXRα and β-catenin expression in the microdissected tissue samples including colorectal cancers, tissue adjacent to and distant normal tissue. As shown in Figure 1 RXRα expression was decreased in colorectal cancer (p=0.0035), adjacent normal (p=0.029), but not in normal tissue distant from the resection site. The β-catenin staining revealed the reverse trend with normal tissue showing the least intensity, while colorectal cancers (p=0.005) and normal tissue adjacent (p=0.001) to cancer stained more intensely. These results suggest that RXRα is commonly under expressed in human colon cancer and is inversely associated with proliferative capacity.

**Figure 2:**
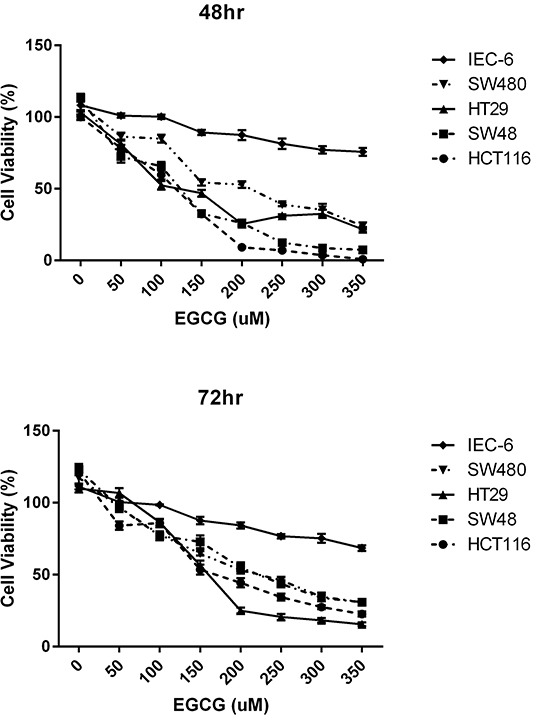
EGCG inhibits proliferation human colon cancer cells **A.** 48 hr treatment time with various EGCG concentrations using CIMP+ lines: HCT116, SW48, HT29; CIMP- SW480 and normal rat epithelial line IEC-6. **B.** 48 hr treatment time with various EGCG concentrations using CIMP+ lines: HCT116, SW48, HT29; CIMP- SW480 and normal rat epithelial line IEC-6.

To further explore the possibility that EGCG could reverse RXRα loss of expression in human colon cancer, we tested the ability of EGCG to suppress proliferation in several human colon cancer cell lines, chosen for differences in their molecular phenotypes. We used lines classified as CpG island methylator phenotype: CIMP+ (HCT116, SW48, HT29) and CIMP- (SW480) [30–33]. CIMP+ cells, unlike CIMP- cell lines, typically demonstrate cancer-specific methylation of key regulatory genes (mainly DNA mismatch repair and tumor suppressors) and exhibit abnormal DNMT activity [34–37]. This difference allowed us to study epigenetic status in relation to RXRα expression or its absence in the human colon cancer cells. Since normal human colon cell lines are relatively unavailable we also tested EGCG cytotoxic effects in IEC-6 cells, a normal rodent colon cell line. As shown in Figure 2, EGCG was generally anti-proliferative to colon cancer cell lines in a time and dose related manner. The CIMP+ cell lines were slightly more sensitive to EGCG treatment, while the IEC-6 line was least responsive.

To further determine how EGCG treatment effects cell growth, we analyzed its effect on cell cycle progression. CIMP+ and CIMP- cell lines were treated with different doses of EGCG (0 - 150 μM) followed by analysis of cell cycle distribution using propidium iodide staining and flow cytometry. Treatment with EGCG promotes cell cycle arrest at G1/S phase in various CIMP+ and CIMP- colon cancer cell lines (Table 1). The effective dose and duration of EGCG treatment that induced cell cycle inhibition varied by cell line. Administration of 50-150 μM EGCG increased the percentage of cells in S-phase in CIMP+ HCT116 cells and CIMP- SW480 cells after 48 hr. EGCG at 100-150 μM resulted in accumulation of cells at G1-phase in CIMP+ SW48 and HT29 cells after 48 hr and after 72 hr at 150 μM (Table 1).

**Table 1:** Cell cycle analysis of CIMP+ (HCT116, SW48, HT29) and CIMP-(SW480) cell lines treated with EGCG or 5-AZA (48 and 72 hr)

48 hr Treatment	% Frequency of Cells					
	HCT116			SW48		
	G1	S	G2	G1	S	G2
EGCG 0 μM	74.48	14.47	9.16	61.3	25.33	6.98
EGCG 50 μM	37.87	38.74	14.47	61.52	23.10	4.56
EGCG 100 μM	50.39	30.04	14.19	77.81	7.60	5.86
EGCG 150 μM	67.94	24.70	7.32	80.44	13.1	0.23
AZA 5 μM	72.74	13.52	11.54	59.25	26.76	7.91

72 hr Treatment	% Frequency of Cells					
	HCT116			SW48		
	G1	S	G2	G1	S	G2
EGCG 0 μM	81.83	11.55	5.21	65.65	22.03	7.01
EGCG 50 μM	43.40	34.87	17.03	73.30	12.69	5.77
EGCG 100 μM	38.56	40.24	11.34	83.24	7.95	4.97
EGCG 150 μM	52.16	33.53	11.42	78.53	10.95	1.95
AZA 5 μM	68.15	20.15	9.26	59.2	25.30	7.80

48 hr Treatment	% Frequency of Cells					
	HT29			SW480		
	G1	S	G2	G1	S	G2
EGCG 0 μM	66.87	19.05	10.01	73.51	13.39	12.19
EGCG 50 μM	50.22	18.16	14.66	53.84	24.75	18.79
EGCG 100 μM	51.87	22.21	7.73	54.56	32.66	9.90
EGCG 150 μM	46.89	20.77	9.33	34.89	52.42	10.27
AZA 5 μM	53.31	26.11	13.67	70.64	14.03	15.77

72 hr Treatment	% Frequency of Cells					
	HT29			SW480		
	G1	S	G2	G1	S	G2
EGCG 0 μM	65.31	11.40	15.99	76.35	12.35	11.08
EGCG 50 μM	62.69	14.30	15.53	63.18	24.63	10.51
EGCG 100 μM	61.58	17.12	11.09	34.42	53.96	7.44
EGCG 150 μM	75.47	12.18	8.94	27.74	60.66	10.26
AZA 5 μM	59.82	15.75	14.66	61.33	19.05	16.49

EGCG treatment induces cell cycle arrest at G1/S phase (highlighted in blue) in various cell lines. (−) No cell cycle fit using Dean-Jett model.

**Figure 3:**
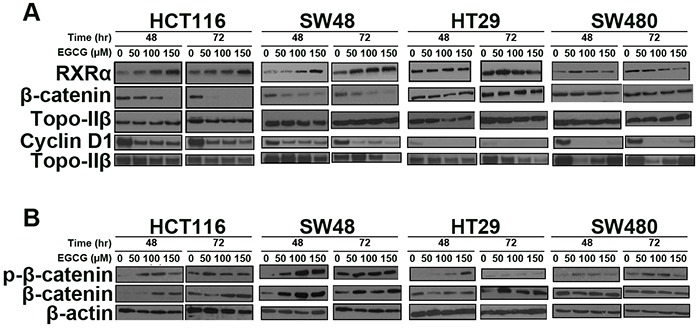
EGCG treatment increases **A.** RXRα protein levels, attenuates nuclear β-catenin levels and cyclin D1 protein levels, and **B.** increase cytosolic phospho-β-catenin and β-catenin in CIMP+ lines (HCT116, SW48), while a modest changes was observed (HT29) and little change in the CIMP- cell line (SW480). Total of 50 μg of nuclear and cytosolic protein was used in each blot.

Using dosing concentrations and time points from the anti-proliferation assays we analyzed protein expression patterns for RXRα, β-catenin and cyclin D1 (Figure 3). In the CIMP+ lines HCT116 and SW48 RXRα protein levels increased with EGCG treatment, with a very modest increase of RXRα in HT29 at 50μM EGCG. Inversely, we observed a decrease in nuclear, free β-catenin protein levels. Subsequently, cyclin D1 protein levels were moderately decreased in the CIMP+ cell lines HCT116 and HT29 treated with EGCG at 48 and 72 hr. The amount of cytosolic β-catenin and phospho-β-catenin protein increased in treated CIMP+ lines. However, in the CIMP- lines the cytosolic β-catenin and phospho-β-catenin protein levels remained relatively unchanged with a slightly lower expression compared to nuclear β-catenin.

**Figure 4:**
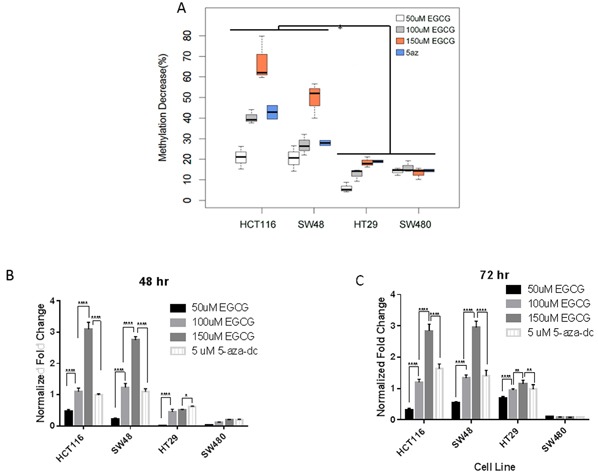
EGCG treatment decreases promoter methylation of RXRα **A.** in CIMP+ lines (HCT116, SW48) with moderate decrease in (HT29). CIMP- lines (SW480) show little change in promoter methylation (p<0.001 HCT116 vs HT29 & SW480, p<0.01 SW48 vs HT29 & SW480). **B&C.** Fold change in RXRα expression in a dose dependent manner and time (48hr & 72hr) dependent manner (**** p<0.001; **p<0.01; *p<0.05).

The RXRα promoter contains several CpG islands that are preferential sites for methylation-induced gene silencing [38]. To determine whether EGCG relieved RXRα promoter methylation we assessed the methylation status of the promoter in CIMP+ and CIMP- cell lines. EGCG decreased the degree of RXRα promoter methylation in CIMP+ cell lines relative to CIMP- lines (Figure 4A). This decrease in methylation also resulted in significant message level increase in RXRα (Figure 4B & 4C), which correlates with the observed restored protein level (Figure 3). Treatment with 150μM EGCG resulted in over 2 fold increase in expression in HCT116 and SW48 cells. The modest decrease in promotor methylation observed in HT29 also correlated with modest increase in expression changes, suggesting that slight changes in methylation correlate in expression within this CpG island in the RXRα promotor. This result confirms that the previously reported methylation decrease in the RXRα promoter and subsequent RXRα RNA transcription increase from the AOM Apc^Min/+^ mouse model given a green tea intervention also applies to certain CIMP+ human colon cancer cell lines [13].

**Figure 5:**
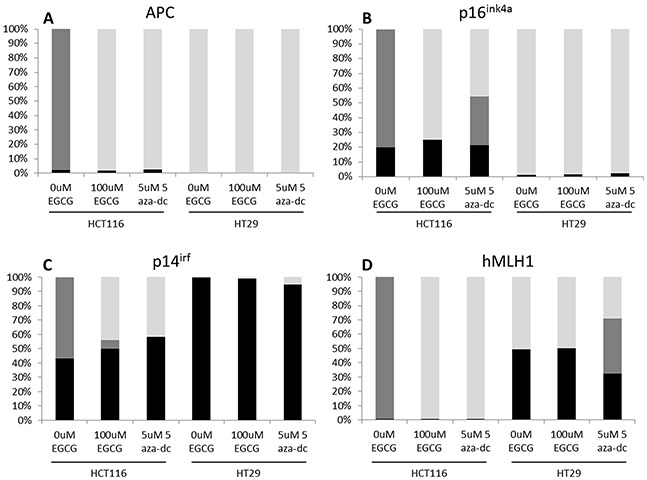
EGCG treatment decreases methylation in the CIMP+ colon cancer cell lines Digested DNA from replicates of biological duplicates were analyzed as described in the methods section. Averages for each cell line x treatment are shown as percent methylation. Black bars: hypermethylated; Gray bars: intermediately methylated; light gray bars: unmethylated.

**Figure 6:**
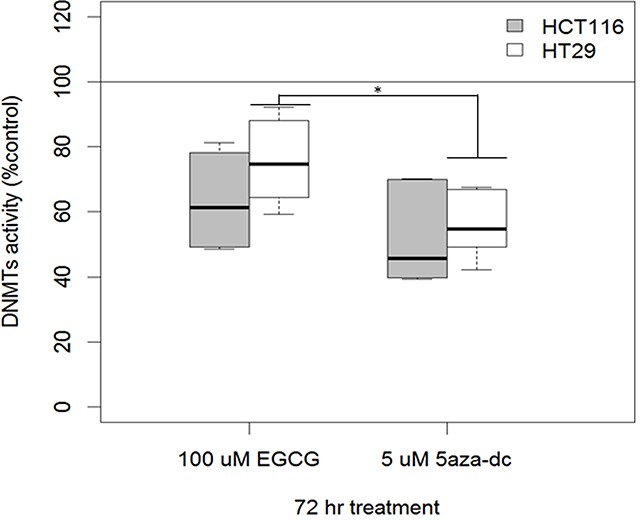
EGCG treatment decreases DNMT activity (DNMT1, DNMT3a, DNMT3b) in human colon cancer cell lines Replicates from biological duplicates are shown (mean ± SE; *p<0.01 5-AZA compared to EGCG – combined cell lines).

To further explore whether EGCG relieved promoter methylation in other target genes reported to be epigenetically silenced in human colon cancer, we examined the degree of methylation in the promoters of *Apc, p14arf, p16ink4a* and *hMLH1* genes in HCT116 and HT29 cell lines. Shown in Figure 5, all four gene promoters in the HCT116 cell line showed a decrease in promoter methylation in response to EGCG treatment, while in the HT29 line there was modest change in promoter methylation. This indicates that EGCG can disrupt methylation silencing in critical genes. Using 5-aza-dc treatment in this assay we found similar changes in demethylation in these four genes. However, not all genes in this assay showed changes in promoter methylation (Table 2), even in our CIMP+ lines. This confirmed that EGCG can repress methylation in certain genes while not inducing a global change in DNA methylation. With disruption of promoter methylation in RXRα and other genes involved in human colon cancers we wanted to determine if EGCG could induce demethylation of DNA by altering protein level and/or activity of methyltransferases. Previous reports have suggested that EGCG can disrupt DNMT1 action by binding to the active pocket [18] and decreasing nuclear protein levels [39]. EGCG treatment of HCT116 showed a marked decrease in total DNMT activity while in HT29 the activity was less affected (Figure 6).

**Table 2:** Methylation changes in the promoters of various genes using the Human Colon Cancer DNA Methylation PCR Array

Gene	Cell Line	0 uM EGCG			100uM EGCG			5uM 5aza-dc		
		Hyp	Int	Non	Hyp	Int	Non	Hyp	Int	Non
**DKK2**	**HCT116**	99.61%	0.00%	0.39%	99.46%	0.00%	0.54%	99.89%	0.00%	0.11%
	**HT29**	98.84%	0.00%	1.16%	98.72%	0.00%	1.28%	98.46%	0.00%	1.54%
**DKK3**	**HCT116**	99.34%	0.00%	0.66%	99.22%	0.00%	0.78%	98.85%	0.00%	1.15%
	**HT29**	99.38%	0.00%	0.62%	99.27%	0.00%	0.73%	98.96%	0.00%	1.04%
**HIC1**	**HCT116**	99.61%	0.00%	0.39%	97.84%	0.00%	2.16%	91.40%	0.00%	8.60%
	**HT29**	3.21%	94.82%	1.97%	5.24%	91.30%	3.46%	3.53%	88.25%	8.22%
**HNF1B**	**HCT116**	99.60%	0.00%	0.40%	55.54%	0.00%	44.46%	50.00%	0.00%	50.00%
	**HT29**	9.73%	0.00%	90.27%	12.31%	0.00%	87.69%	13.21%	0.00%	86.79%
**HS3ST2**	**HCT116**	99.78%	0.00%	0.22%	91.53%	0.00%	8.47%	84.66%	0.00%	15.34%
	**HT29**	17.71%	81.00%	1.28%	30.04%	68.23%	1.73%	17.64%	66.41%	15.95%
**OPCML**	**HCT116**	99.01%	0.00%	0.99%	96.93%	0.00%	3.07%	96.18%	0.00%	3.82%
	**HT29**	98.37%	0.00%	1.63%	98.53%	0.00%	1.47%	97.98%	0.00%	2.02%
**PCDH10**	**HCT116**	99.23%	0.00%	0.77%	98.27%	0.00%	1.73%	95.49%	0.00%	4.51%
	**HT29**	98.99%	0.00%	1.01%	97.72%	0.00%	2.28%	92.45%	0.00%	7.55%
**RASSF1**	**HCT116**	1.69%	98.28%	0.03%	0.59%	0.00%	99.41%	1.27%	0.00%	98.73%
	**HT29**	0.08%	63.43%	36.49%	0.31%	51.84%	47.85%	0.72%	69.66%	29.62%
**RUNX3**	**HCT116**	98.84%	0.00%	1.16%	99.19%	0.00%	0.81%	98.96%	0.00%	1.04%
	**HT29**	99.16%	0.00%	0.84%	97.07%	0.00%	2.93%	99.01%	0.00%	0.99%
**SFRP1**	**HCT116**	99.48%	0.00%	0.52%	96.75%	0.00%	3.25%	89.96%	0.00%	10.04%
	**HT29**	98.29%	0.00%	1.71%	97.45%	0.00%	2.55%	86.42%	0.00%	13.58%
**SFRP2**	**HCT116**	99.62%	0.00%	0.38%	98.61%	0.00%	1.39%	97.77%	0.00%	2.23%
	**HT29**	99.41%	0.00%	0.59%	98.75%	0.00%	1.25%	98.48%	0.00%	1.52%
**SPARC**	**HCT116**	99.26%	0.00%	0.74%	98.41%	0.00%	1.59%	97.11%	0.00%	2.89%
	**HT29**	99.00%	0.00%	1.00%	98.97%	0.00%	1.03%	95.66%	0.00%	4.34%
**TMEFF2**	**HCT116**	99.98%	0.00%	0.02%	99.86%	0.00%	0.14%	−--^1^	−--	−--
	**HT29**	10.99%	88.97%	0.03%	99.61%	0.00%	0.39%	99.80%	0.00%	0.20%
**UCHL1**	**HCT116**	99.99%	0.00%	0.01%	99.67%	0.00%	0.33%	99.73%	0.00%	0.27%
	**HT29**	99.87%	0.00%	0.13%	99.56%	0.00%	0.44%	99.88%	0.00%	0.12%
**WIF1**	**HCT116**	99.57%	0.00%	0.43%	98.96%	0.00%	1.04%	99.26%	0.00%	0.74%
	**HT29**	99.78%	0.00%	0.22%	99.42%	0.00%	0.58%	99.09%	0.00%	0.91%
**WT1**	**HCT116**	100.00%	0.00%	0.00%	98.97%	0.00%	1.03%	98.62%	0.00%	1.38%
	**HT29**	99.11%	0.00%	0.89%	99.52%	0.00%	0.48%	49.17%	50.09%	0.74%

1 No results were obtained for this sample

## DISCUSSION

In this study we establish that CIMP+ human colon cancer cell lines demonstrate reduced expression of the nuclear transcription factor RXRα and expression of this gene was restored using EGCG, a classic SMNP, which reduced the degree of promotor methylation in this gene. Epigenetic silencing of key regulatory genes appears to be a common event in CIMP+ colon cancers [1, 3, 6, 34, 40]. Because of the reversible nature of epigenetic changes, it is possible that de-silencing of “silenced” genes in cancer could restore a semblance of control, and lead to suppression of cancer [2, 3, 5, 6, 41]. A number of SMNPs aside from EGCG are known epigenetic regulators: apigenin, folate, genistein, lycopene, myricetin, naringenin, phloretin, protocatechuric acid, quercetin, rosmarinic acid, sinapinic acid and sulforaphane; their utility as cancer preventives in this context is the subject of current exploration [6].

Methylation of the promoter of RXRα is one mechanism in which colon cancer tumors disable a key regulatory network. RXRα is a major heterodimerization partner with LXR [27], FXR, RAR, PPAR and VDR [26, 42]. The dimerization of RXRα and VDR is critical and when interrupted through epigenetic silencing or polymorphism the functions of VDR can be disrupted. Many genes contain vitamin D response elements and a large number of these are associated with control of inflammation, an important aspect in the initiation, progression, and late stage colon carcinogenesis [26, 42]. Thus impairment of RXRα either by epigenetic silencing or mutation could impact on the response of transcriptional machinery dictated by specific response elements in genes associated with progression or inhibition of cancer and present important targets for chemoprevention. This is a different approach compared to using drugs to enhance expression, such as the RXRα agonist Bexarotene [43–45]. In this study we show that EGCG inhibited promoter methylation of RXRα resulting in a restoration of RXRα gene expression and protein levels. Several mechanisms are involved in the release of silenced genes such as RXRα. Our data suggests that EGCG treatment of CIMP+ colon cancer cell lines is accompanied by a reduction in DNMT expression. Although the effective doses of 50-100μM EGCG, in short duration, used in this study are attainable *in vitro* but may not be biologically achievable in humans [46, 47], they are instructive in assessing possible mechanisms of compounds such as EGCG on epigenetic mechanisms and biological markers. Within this field there is always considerable hesitation that *in vitro* EGCG effects will be observed in vivo (although we have shown that EGCG does have these effects in animal models), as goes with many natural products [13]. There are several studies which suggest that therapeutic applications of EGCG above 50uM are optimal and achievable [48–51]. There is promising research to suggest that EGCG can inhibit DNMT activity in *in vitro* assays at much lower concentrations than used in this study [18, 19]. Future experiments using lower, more biologically relevant doses over longer periods will need to be conducted to validate these results, as well as *in vivo* studies utilizing xenograft models and RXRα haplodeficient mice [52, 53]. Bioavailability of EGCG and green tea components (as well as other plant flavonoids) is a critical issue to address in murine models of colorectal cancer but our results emphasize that certain SMNPs like EGCG do have epigenetic modulatory promise.

Numerous signaling pathways have been identified in cancer and studied as targets for therapies. The pleoitropic effects of SMNPs like EGCG make them interesting candidates as both preventive and therapeutic agents. Another benefit of SMNPs is the ease of delivery via oral exposure, fewer side effects, and ability to be used over longer periods of time, as voiced by Sporn, features of the ideal preventive agent [54]. The discovery that certain SMNPs can act as chemoprevention agents, through epigenetic regulation has sparked a growing interest in cancer biology research [6, 9, 13, 28, 34]. Green tea is one natural source that contains a hypothesized epigenetic regulator in EGCG. In mouse and human colon cancer cells, EGCG has been shown to reduce DNA methylation in numerous genes [5, 12, 16, 22, 35, 38, 40, 55]. Silencing of RXRα could be a sentinel event in some human cancers. Specifically RXRα has been identified to be silenced by genetic mutations in human colon cancer and is associated with risk for colon adenoma, thus this inactivation, either by polymorphism or by silencing could be an important early event in carcinogenesis [25].

In summary, we found that RXRα expression was decreased in malignant human colorectal tumors. In CIMP+ human colon cancer cell lines we found RXRα promoter methylation is modulated by EGCG. Subsequently this lead to a decrease in nuclear β-catenin and cyclin D1, disruption of cell proliferation, and promotion of cell cycle arrest. A translational limitation of our study on RXRα status in human tumor specimens was the use of a commercial tissue microarray. While we demonstrated downregulation of RXRα expression in the colorectal cancer samples in the array, we did not have access to pertinent clinical information to correlate tumor phenotype with impaired RXRα expression and possible associations with CIMP+ status. The exact nature of EGCG as an epigenetic regulator, either as a DNMT inhibitor (or HDAC inhibitor), or both may be a function of dose and time of exposure [16, 17, 24, 39, 55]. Silencing of RXRα implies impairment of a number of key transcription factors involved in carcinogenesis, primarily among these is the vitamin D receptor [4, 10, 19, 34, 56]. Studies in our laboratory continue to identify whether silencing of RXRα and its implication for loss of regulation of inflammation are central to the development of colon cancer and it's potential as target for prevention.

## MATERIALS AND METHODS

### Source of reagents

EGCG (E4143) and 5aza-dc (A3656) were purchased from Sigma-Aldrich (St. Louis, MO, USA)

### IHC of human colon tissue microarray

Human colon tissue microarray (CO1002) slides were purchased from US Biomax, Inc. (Rockville, MD, USA). IHC was done as previously reported [13] using RXRα and β-catenin antibodies (Santa Cruz Biotech, Santa Cruz, CA, USA). Immunohistochemical analysis: The stained slides were scanned using a digital slide scanner system (Olympus America Inc, Center Valley, PA, USA) comprised of a BX61TRF5 Olympus microscope frame with a motorized stage controlled by software and a DP71 12 MP digital color camera. Each spot of the tissue microarray was completely scanned at 100X magnification. Analysis of both intensity and area of positive (brown) staining was performed using the Visiomorph Image Analysis Software. An image classification system was used to convert the Red-Green-Blue (RGB) channels of the original image into a Red-Green contrast. The software was calibrated to recognize the following areas in each spot: background (represented by areas of glass slide without any tissue on it), negative (represented by areas counterstained with hematoxylin but not stained in brown by DAB), and positive (represented by areas stained in brown by DAB). The outcome measures of interest for each individual spot extracted from the software were: area of positive staining, area of negative staining, total area (area of positive staining+area of negative staining) and intensity of positive staining. We analyzed the data using the fraction of positively-stained area (area of positive staining/total area of the spot) and the normalized intensity of positive staining (intensity of positive staining/area of positive staining).

### Cells and growing conditions

All cell lines (HCT116, HT29, SW48, SW480, IEC-6) were purchased from ATCC (Manassas, VA, USA) within 6 months of the experiments described. Cells were grown in McCoys 5A (supplemented 10% FBS and 1%Pen/Strep at 37°C, 5% relative CO_2_) for HCT116 and HT29. For SW48 and SW480 cells were grown using L-15 (supplemented with 10% FBS and 1%Pen/Strep at 37°C). IEC-6 cells were grown in Eagle's media (with 10% FBS and 1%Pen/Strep at 37 C°, 5% relative CO_2_). All cell culture media was purchased from Cellgro (Manassas, VA, USA).

### MTS cell viability assay: effects of EGCG treatment

Cells were plated in 96 well plates at the following densities: 5,000/well for HCT116, SW48, and SW480; 10,000/well for HT29 and IEC-6). All cells were allowed to adhere overnight in their respective complete media. Cells were serum starved overnight and then treated with the appropriate concentration of vehicle (DMSO (0 EGCG)), EGCG (50, 100, 150, 200, 250, 300, 350 μM) or 5 μM 5aza-dc (Sigma-Aldrich, St. Louis, MO, USA) or no vehicle/EGCG treatment to serve as control of cellular growth. After 48 and 72 hr, 12 μl of viability reagent was added and allowed to react for 3 hr (CellTiter 96^®^ AQ_ueous_ Non-Radioactive Cell Proliferation Assay (MTS); Promega, Madison, WI, USA). Cells were grown in triplicates with two biological replicates for each cell line/treatment/time point. Percent viability was calculated using no vehicle/EGCG cells as 100% viable normalization reading.

### Cell cycle analysis

Cells were plated in six-well culture plates (500,000 cells/well) for 24 hr under standard growth conditions, followed by serum starvation for 24 hr. Cells were treated with EGCG (0, 50, 100, 150 μM) or 5 μM 5aza-dc for 48 and 72 hr. Subsequently, cells were trypsinized, centrifuged at 1400 rpm (4°C, 5 min), washed twice with PBS, fixed in 70% ethanol (1×10^6^ cells/tube), and stored at −20°C. Fixed cells were centrifuged at 1400 rpm (4°C, 5 min) and washed with PBS. Cells were resuspended in 350 μL PBS containing 10 μg/mL propidium iodide (Sigma, St. Louis, MO, USA) and 1 μg/mL RNase A (St. Louis, MO, USA) and stained for 30 min at room temperature. Data were acquired using a FACSCalibur flow cytometer (BD BioSciences, San Jose, CA, USA) and analyzed using FlowJo version 7.6.5 software (Tree Star, Inc., Ashland, OR, USA). Singlets were gated and doublets were discriminated in all samples.

### Western blot analysis

Treated cells (0, 50, 100, 150 μM EGCG at 48 and 72 h) were plated at higher densities (~100,000) in 100 mm^3^ plates. Cytosolic and nuclear protein were isolated by washing with cold PBS, lysed with 750 μL cold low salt buffer (10mM HEPES, 10mM KCl, 1mM EDTA, and protease inhibition tabs – Roche Scientific, Pleasanton, CA, USA) for 10 min on ice. Subsequently, 25μL 10% NP40 was added, cells were spun at 6000 rpm for 6 min, the supernatant was removed (cytosolic protein fraction) and stored at −80°C. The remaining pellet was lysed with high salt buffer (20MM HEPES, 0.4M NaCl, 1mM EDTA, protease inhibition tabs) on ice for 30 minutes. Cells were spun at 13,000 rpm for 10 min and the supernatant was removed (nuclear fraction) and stored −80°C. Protein concentrations were read on a NanoVue (GE Healthcare, Piscataway, NJ, USA). Nuclear protein (50μg) or cytosolic protein (50μg) were run on 12% polyacrylamide gel and transferred to PVDF membranes. Primary antibodies (RXRα sc-553 1:250, β-catenin sc-7963 1:500, Topo IIβ sc-13059 1:500, Santa Cruz Biotech, Santa Cruz, CA, USA, anti-phospho-β-Catenin (Ser37): 07-1651 1:500, Millipore, Billerica, MA, USA, anti-Cyclin D1 [SP4] ab16663 1:200, anti-TOPO IIβ [EPR5377] ab109524 1:1000, Abcam, Cambridge, MA, USA) were incubated overnight followed by the appropriate secondary for 3 h. Chemiluminescent detection was done using Supersignal West Pico Kit (Thermo Scientific, Rockford, IL, USA).

### RXRα qPCR

Cell were treated with EGCG at 0, 50, 100, 150 μM EGCG for 48 hr and 72 hr. Total RNA was isolated using Aurum total extraction kit (Bio Rad, Carlsbad, CA, USA) and first cDNA created using iScript cDNA synthesis kit (Bio Rad, Carlsbad, CA, USA). The iQ SYBR supermix (100ng of cDNA) was utilized to measure RXRα levels with the following primers: forward 5′ TCCACCCAGGTGAACTCCTCCC 3′; reverse 5′ GGTGGGCACCGACATGGAGTG 3′, β-actin forward 5′ TGAGCGCGGCTACAGCTT 3′; reverse 5′ TCCTTAATGTCACGCACGATTT 3′. Relative fold changes were calculated comparing ΔΔCT values using β-actin as the normal expression target.

### RXRα promoter methylation

Treated cells (0, 50, 100, 150 μM EGCG for 72 h) were plated at higher densities (~100,000) in 100 mm^3^ plates. After treatment DNA was isolated using DNeasy kit (Qiagen, Valencia, CA, USA). DNA was bisulfite treated using an Imprint DNA Modification Kit (Sigma-Aldrich, St. Louis, MO, USA) and subsequently DNA sequenced (Genewiz, Southplains, NJ, USA). A 600 bp region of the RXRα promoter from −1 to −600 of the start codon was amplified from treated and control cells using the following primers F: 5′ GGAGCTTGTCCTCTGCCGTTGGGG 3′; R: 5′ GTCTGCGACTAACTCATGCCCGGC 3′. Sequences from −360 to −480 were compared to measure changes in methylated cytosines.

### Methylation array

Isolated DNA from treated cells (as described previously) were digested using SA Bioscience EpiTech Methyl DNA Restriction Kit (Qiagen, Valencia, CA, USA) following the manufacturer's directions. The digest was analyzed for methylation changes using Human Colon Cancer DNA Methylation PCR Array (MeAH-9060, Qiagen, Valencia, CA, USA).

### DNMT inhibition assay

Protein was isolated from treated cells (as previously described) using EpiQuik Nuclear Extraction Kit II (Epigentek, Farmingdale, NY, USA) following the manufacturer's directions. DNMT inhibition was analyzed using EpiQuik DNA Methyltransferase Activity/Inhibition Assay Kit (P-3001-2, Epigentek) following the manufacturer's directions.

### Statistical analysis

P-values were calculated using Wilcoxon rank sum tests for pairwise comparison of conditions. For Figure 1, some malignant and adjacent samples were paired (i.e., from the same patient) and so a standard approach for comparing mean expression (such as the t-test) is not valid. To account for the lack of independence, a random effects linear regression model was used where random intercepts were included. Because the expression ratio data distribution was highly right-skewed, the log was taken which symmetrized the data before performing the regression. Wald tests of regression coefficients (and their differences) were used to evaluate statistical significance of differences in expression across the three groups. For Figure 5, linear regression was used to compare the fraction of hypermethylated cells in the 100 uM ECGC vs. no treatment and 5-AZA vs. no treatment. This comparison was repeated for intermediate cells.
